# Association between the Systemic Immune-Inflammation Index and Thyroid Function in U.S. Adults

**DOI:** 10.1155/2023/5831858

**Published:** 2023-11-16

**Authors:** Xin-Yu Hu, Ying-Chao Liang, Huan-Huan Zhang, Hui-Lin Li, De-Liang Liu

**Affiliations:** ^1^The Fourth Clinical Medical College of Guangzhou University of Chinese Medicine, Shenzhen, Guangdong, China; ^2^Department of Endocrinology, Shenzhen Traditional Chinese Medicine Hospital, Shenzhen, Guangdong, China

## Abstract

**Background:**

The systemic immune-inflammation index (SII) is used as an indicator of prognosis for a wide range of diseases. Thyroid function has been found to be strongly associated with inflammation. The purpose of this investigation was to analyze the correlation between SII and various thyroid functions.

**Methods:**

This study utilized data from the National Health and Nutrition Examination Survey (NHANES) 2007–2012. The association between SII and thyroid function was analyzed using weighted univariate and multivariate linear regression analyses. Subgroup analyses, interaction tests, and weighted restricted cubic spline (RCS) regression analyses were also employed to test this correlation.

**Results:**

Of the 6,875 participants (age ≥ 20 years), the mean age was 46.87 ± 0.40 years. The adjusted model showed that lnSII was negatively correlated with FT3 (*β* = −0.0559, 95% CI −0.1060 to −0.0059,) and FT3/FT4 (*β* = −0.0920, 95% CI −0.1667 to −0.0173,). There was a positive correlation between lnSII and TT4 (*β* = 0.1499, 95% CI 0.0722–0.2276,). In subgroup analyses, lnSII still independently affected a wide range of thyroid functions. Weighted RCS analysis showed a nonlinear relationship between FT3 and lnSII.

**Conclusion:**

Close relationships exist between SII and a variety of thyroid functions. SII can be used as an indicator to predict thyroid dysfunction. Control of inflammatory activity may be a protective measure against thyroid dysfunction. More large-scale prospective studies are necessary to further explore the correlation between SII and thyroid function and the role of obesity in this.

## 1. Introduction

Triiodothyronine (T3) and tetraiodothyronine (T4) are the two major forms of thyroid hormones that are released by the thyroid gland and are regulated by the hypothalamic–pituitary–thyroid axis [1]. The synthesis and secretion of thyroid hormones are stimulated by thyroid stimulating hormone (TSH), which is synthesized by the adenohypophysis. T3 and T4 secreted into the circulation are tightly bound to plasma proteins, a small proportion of these that are biologically active are known as free triiodothyronine (FT3) and free thyroxine (FT4), respectively [2]. Thyroglobulin antibodies (TgAb) and thyroid peroxidase antibodies (TPOAb) are related to autoimmune thyroid disease and are commonly used to identify patients at high risk of progressing to hypothyroidism requiring treatment [3].

Thyroid hormones are crucial in various physiological processes, including growth, normal development, neural differentiation, and metabolic regulation. It can regulate energy expenditure and thermogenesis, the composition of lipids, and ensure the maintenance of normal reproductive function [4, 5]. Fluctuations in thyroid hormone have been correlated with a variety of diseases, an excess of thyroid hormone may lead to atrial fibrillation and heart failure [6, 7]. Insufficient thyroid hormone is associated with a high risk of hypertension, dyslipidemia, and type 2 diabetes [8, 9]. Due to the significant negative impact of adverse clinical outcomes from thyroid dysfunction, a high degree of clinical attention should be paid to changes in thyroid function.

Previous studies have found that abnormal thyroid function is closely related to the inflammatory response. Large population-based studies have revealed a potential association between systemic inflammation and the formation and progress of thyroid nodules, and inflammatory markers are strongly correlated with the size of thyroid nodules in all genders [10]. COVID-19-mediated overproduction of inflammatory cytokines like IL-6, IL-1*β*, and interferon-*γ* increased the incidence of subacute thyroiditis, Graves' disease, and Hashimoto's thyroiditis, which affect thyroid function [11]. Animal studies have found that inflammatory vesicle activation in obese mice upregulates deiodinase activity by downregulating ubiquitin-mediated protein degradation. This contributes to thyroid hormone resistance at the pituitary level and has an effect on thyroid hormones [12, 13].

The systemic immune-inflammation index (SII) is widely recognized as a stable and comprehensive indicator of the body's local immune response and systemic inflammatory state [14, 15]. SII is a composite index based on peripheral blood lymphocyte, neutrophil, and platelet counts (SII = platelet counts × neutrophil/lymphocyte counts). Several previous studies have shown that SII can predict the prognosis of tumor patients by reflecting the balance between the body's inflammatory and immune responses and has high prognostic value for patients with prostate cancer [16], hepatocellular carcinoma [14], cervical cancer [17], and biliary tract cancer [18]. SII provided more clinical information than previous inflammatory factors. Patients with high levels of SII were associated with thrombocytosis, neutrophilia, or lymphocytopenia [19]. It is now widely recognized that SII accurately assesses the inflammatory balance of the body [20], which reflects the degree of systemic inflammatory activity, with a higher SII representing a more pronounced inflammatory response. A higher SII was found to be correlated with an increased risk of type 2 diabetes mellitus combined with depression [21], urinary albumin excretion [22], osteoporosis in postmenopausal women [23], peripheral arterial disease [24], ulcerative colitis [25], and diabetic nephropathy [20]. However, there are no studies on SII and thyroid function at this time.

To fill this gap, the objective of this study was to explore the correlation between SII and thyroid function using a nationally representative sample of adults (age ≥ 20 years) in the United States.

## 2. Materials and Methods

### 2.1. The Subjects in the Study

Subject data for this study were obtained from the National Health and Nutrition Examination Surveys (NHANES). NHANES is a nationwide, population-based, and cross-sectional survey dedicated to collecting information on the nutritional status and health risk factors of U.S. nationals. NHANES incorporates nationally representative subjects through a well-designed, complex, stratified, and multistage sampling design. Informed permission was obtained in writing from all participants before they were enrolled in the investigation. The study design and written informed consent were examined and confirmed by the Research Ethics Review Board of the National Center for Health Statistics (NCHS). Specific research programs and information are available at https://www.cdc.gov/nchs/nhanes/.

**Figure 1:**
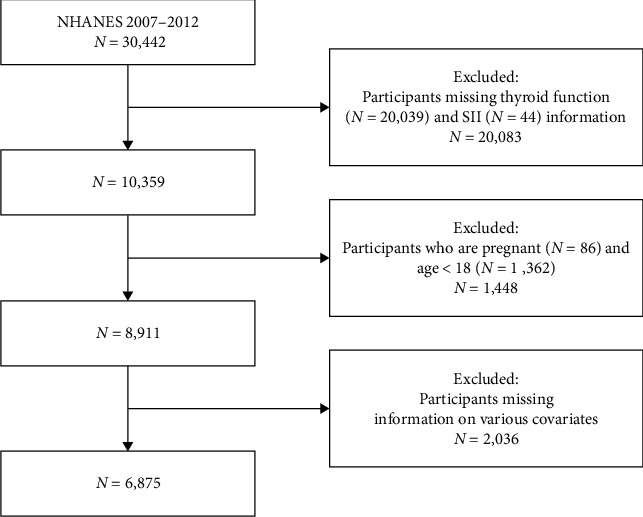
Flowchart of the participants' selection from NHANES 2007–2012.

Three cycles of data from the database, 2007–2008, 2009–2010, and 2011–2012, were included in this study. We excluded from the 30,442 subjects 20,039 participants missing relevant thyroid function information, 44 participants missing SII data, 1,362 participants younger than 18 years of age, and 86 pregnant patients. This was followed by the exclusion of 430 participants missing information on marital status and education level, 759 missing information on family poverty income ratio (PIR), 100 missing information on body mass index (BMI), 575 missing information on alcohol use and smoking status, 23 missing information on glycosylated hemoglobin and alanine aminotransferase (ALT), and 149 missing information on urine iodine concentration (UIC). Since NHANES only collected data related to smoking and alcohol consumption for people 20 years of age and older, we excluded people under 20 years of age from the subject exclusion process. Ultimately, 6,875 U.S. adult participants aged 20 years or older were enrolled in the study (Figure 1).

### 2.2. Systemic Immune-Inflammation Index

As the independent variable in this study, SII was calculated on the basis of a complete blood count test. According to Hu et al. [14], the definition of SII is as follows: SII = platelet counts × neutrophil/lymphocyte counts, which was presented as ×10^9^ cells/*μ*L [22]. Complete blood counts were analyzed using automated hematology analysis instruments (Coulter® DxH 800 analyzer). Detailed laboratory methodology can be found on the NHANES website.

### 2.3. Thyroid Function

Thyroid function was the dependent variable in this study, which in this study included FT3, FT4, TT3, TT4, FT3/FT4, TSH, TG, TGAb, and TPOAb. Competitive binding immunoenzyme assays were used to measure serum TT4, TT3, and FT3, while two-step enzyme immunoassays were used to measure serum FT4. Using a two-site immunoenzymatic test, serum TSH levels were determined. The FT3/FT4 value was calculated to reflect the activity of peripheral T4 to T3 conversion [26]. More detailed collection and processing information can be viewed in the NHANES Laboratory/Medical Technologists Procedures Manual.

### 2.4. Covariates

A number of covariates potentially associated with SII and thyroid function were preselected and collected from the NHANES. Demographic covariates included age, sex, race, marital status, education level, and family PIR, which were collected through in-person family interviews. Lifestyle habit-related covariates included smoking status, alcohol use, and BMI, which were obtained from health questionnaires. Clinically relevant index covariates included UIC, glycosylated hemoglobin (HbA1c), ALT, aspartate aminotransferase (AST), creatinine (Cr), total cholesterol (TC), and the previously mentioned thyroid function, which were derived from analyses of laboratory instruments. Preexisting disease-related information, including the presence of diabetes, was obtained through questionnaires and laboratory tests. The diagnosis of diabetes in this study was made through the following criteria: (1) the participant had been diagnosed with diabetes by a physician, (2) glycosylated hemoglobin was greater than 6.5%, (3) fasting blood glucose was greater than 7 mmol/L, (4) random blood glucose or 2-hr postprandial blood glucose was greater than or equivalent to 11.1 mmol/L, and (5) insulin or diabetic medication was being used. According to the guideline published by the American Diabetes Association [27], a diagnosis of diabetes is confirmed when any of these criteria are met.

For some continuous variables, we did some regrouping. The family PIR was classified as <1, ≥3, and ≥1, <3 to assess participants' socioeconomic status. Alcohol use was categorized according to the frequency of drinking: never: had <12 drinks in their entire lives; former: drank 12 or more times in 1 year but abstained from alcohol last year, or abstained from alcohol last year but drank 12 or more times in their entire lives; mild: <2 drinks/day; moderate: ≥2 drinks/day for women, ≥3 drinks/day for men, and ≥2 day/month for those who binge drink; and heavy: 3 drinks/day for women, ≥4 drinks/day for men, or binge drinking ≥5 days/month. Smoking status was categorized according to the frequency of smoking: never: smoked fewer than 100 cigarettes in his or her lifetime; former: consumed over 100 cigarettes in his or her lifetime and has not smoked any until now; and now: consumed over 100 cigarettes in his or her lifetime and now smoke sometimes or daily. According to WHO [28], BMI was categorized into four levels: (1) ≤18.5 kg/m^2^, (2) >18.5 and ≤25.0 kg/m^2^, (3) >25.0 and ≤30.0 kg/m^2^, and (4) >30 kg/m^2^. Urinary iodine concentrations were categorized into three groups: (1) <100 *µ*g/L, (2) ≥100 and <300 *µ*g/L, and (3) ≥300 *µ*g/L.

### 2.5. Statistical Analysis

The statistical analyses in this study followed the recommendations of the CDC guidelines. Sampling weights were employed in the statistical analyses given the complex, multistage sampling design of the NHANES data collection. Means and standard error of mean were reported for continuous variables, whereas percentages and 95% confidence intervals (CIs) were presented for categorical variables. Utilizing survey-weighted linear regression analysis to test the differences between three SII tertile groups for continuous variables and survey-weighted chi-square testing for categorical variables. Due to the skewed distribution of the SII data, we performed a natural log transformation on SII and divided it into three groups by thirds tertiles. We first analyzed the relationship between lnSII and each thyroid function using weighted univariate linear regression analysis. Thyroid functions with statistically significant associations with lnSII were selected. We then used weighted multivariate linear regression to analyze the connection between lnSII and selected thyroid functions. To further analyze the impact of covariates on these associations, we established three models according to the recommendations of Strengthening Reporting of Observational Studies in Epidemiology (STROBE). Model 1 did not make any covariate adjustments, Model 2 adjusted for age, sex, race, marital status, family PIR, and education level, and Model 3 adjusted for all covariates. Considering the effects of BMI, UIC, and sex on thyroid function as well as conducting the sensitivity analyses [29–31], we performed subgroup analyses with BMI, UIC, and sex subgroups. A weighted analysis was used in the subgroup analyses and adjusted for all covariates. To test for heterogeneity between subgroups, we performed interaction term tests. Last, we analyzed the nonlinear relationship between lnSII and thyroid function and performed sensitivity analyses using weighted restricted cubic spline (RCS) regression analyses with four slice points for modeling. R software was used for all statistical analyses, and a two-tailed *p*-value of less than 0.05 was considered significant.

## 3. Results

### 3.1. Baseline Characteristics of Participants

This study included 6,875 participants from NHANES 2007–2012, of which 3,424 (49.81%) were male and 3,451 (50.19%) were female. Among all participants, the mean age was 46.87 ± 0.40 years, and 4,932 (71.74%) were non-Hispanic White. After using sample weights, the 6,875 participants represented 9,104,2939 noninstitutionalized citizens in the United States. As demonstrated in Table 1, thyroid functions with statistically significant differences (*p* < 0.05) between SII tertiles included FT3, FT4, TT4, FT3/FT4, and TGAb. The ranges of the three SII tertiles were, respectively, 13.750–387.529, 387.545–596.077, and 596.111–4,085. Among the covariates, the variables with statistically significant differences were sex, race, marital status, family PIR, smoking status, BMI, AST, and TC (*p* < 0.05).

### 3.2. The Association between Systemic Immune-Inflammation Index and Thyroid Function

We first employed the weighted univariate linear regression analysis to analyze the connection between lnSII and thyroid function (Table 2). The association between FT3, TT4, FT3/FT4, TG, TPOAb, and lnSII was found to be statistically significant (*p* < 0.05) when each thyroid function was included in a univariate regression model separately.

We then included FT3, TT4, FT3/FT4, TG, and TPOAb in the weighted multivariate linear regression for analysis, respectively. The independent connection between lnSII and thyroid function was also evaluated using a variety of models. In Table 3, the outcomes of the weighted multivariate linear regression analysis are displayed. In the unadjusted model, lnSII was negatively correlated with FT3 (*β* = −0.0603, 95% CI −0.1078 to −0.0128, *p* < 0.05) and FT3/FT4 (*β* = −0.0918, 95% CI −0.1658 to −0.0178, *p*=0.019). There was a positive correlation observed between lnSII and TT4 (*β* = 0.1837, 95% CI 0.0974–0.2700, *p* < 0.001) and TPOAb (*β* = 5.1330, 95% CI 0.4263–9.8398,). The relationships between FT3 (*β* = −0.0559, 95% CI −0.1060 to −0.0059, *p*=0.039), FT3/FT4 (*β* = −0.0920, 95% CI −0.1667 to −0.0173, *p*=0.024), TT4 (*β* = 0.1499, 95% CI 0.0722–0.2276,), and lnSII also remained significant in the fully adjusted model.

After grouping lnSII by tertiles, lnSII remained negatively correlated with FT3 and FT3/FT4 and positively correlated with TT4. There was no statistically significant link between lnSII and TG (*β* = 1.6395, 95% CI −0.1964 to 3.4754, *p*=0.093) when presented as a continuous variable, but its effect size was more evident and statistically significant when presented as a categorical variable (Tertiles 3, *β* = 3.1108, 95% CI, 0.2400–5.9817, *p*=0.045). Continuous and categorical variables of lnSII and TPOAb were only statistically significant in the crude model, and the association between TPOAb and lnSII was not statistically significant after adjusting for confounders.

### 3.3. Subgroup Analysis

We conducted three subgroup analyses to examine the association between SII and thyroid function in various populations that were categorized according to different gender, BMI, and UIC, and tested the significance of the interaction term with exposure. Analysis grouped according to gender showed that the relationship between lnSII and FT3, TT4, FT3/FT4, and TG remained consistent in different genders (Table 4). The relationship between lnSII and FT3, FT3/FT4, and TG was more pronounced in the female population compared to the male and general population. There was an interaction between the association of lnSII and FT3 in the sex subgroups (*p* < 0.05). In subgroup analysis grouped according to BMI, the relationships between FT3, TT4, FT3/FT4, and lnSII remained generally consistent between the different BMI groups but were all statistically significant only in the group of people with their BMI > 25.0 and ≤30.0 kg/m^2^ (Table 5). TG was positively associated with lnSII in those with their BMI >30 kg/m^2^ and the effect size was more significant (*β* = 5.1916, 95% CI 1.9946–8.3887,). Interaction tests suggested an interaction between FT3, FT3/FT4, and TG in the BMI subgroups (*p* < 0.05). In subgroup analysis stratified by UIC, the relationships between FT3, TT4, FT3/FT4, and lnSII remained consistently the same (Table 6). Nevertheless, these relationships were statistically significant only in the population with their UIC ≥100 and <300 *µ*g/L. There was an interaction between the association of lnSII and FT3 in the UIC subgroups (*p* < 0.05). As part of the sensitivity analysis, the results of the subgroup analyses indicated that the results of this study were very robust.

### 3.4. Weighted Restricted Cubic Spline Regression Analysis

**Figure 2:**
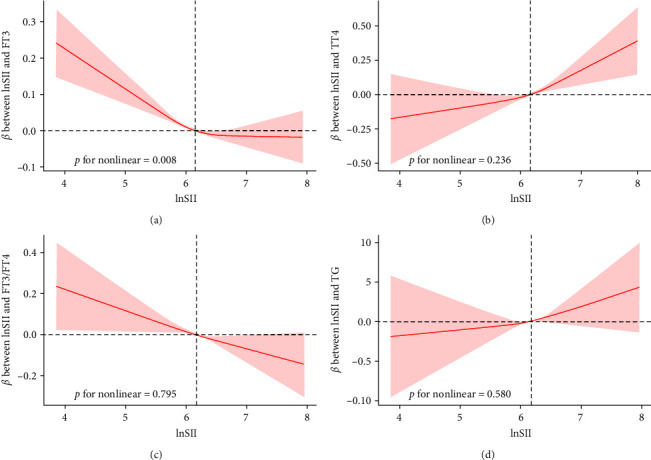
Weighted RCS analysis of the association between lnSII and thyroid function. (a) RCS curve of the association between FT3 and lnSII; (b) RCS curve of the association between TT4 and lnSII; (c) RCS curve of the association between FT3/FT4 and lnSII; and (d) RCS curve of the association between TG and lnSII. The model was adjusted for age, sex, race, marital status, family poverty income ratio, education level, body mass index, smoking status, alcohol use, diabetes, glycated hemoglobin, alanine aminotransferase, aspartate aminotransferase, creatinine, urine iodine concentration, and total cholesterol.

To further discuss the nonlinear relationship between SII and thyroid function, we performed a weighted RCS analysis (Figure 2). After adjusting for confounders, the results of the weighted RCS analysis showed a negative correlation with FT3 with lnSII, and this correlation followed a nonlinear pattern (*p* for nonlinear = 0.008). A threshold effect can be observed. As shown in Figure 2(a), when lnSII is less than about 6.17, the curve declines rapidly with the increase of lnSII, and when lnSII is greater than about 6.17, the decline of the curve is relatively smooth. Weighted RCS analysis showed no statistically significant nonlinear relationship between TT4, FT3/FT4, TG, and lnSII (*p* > 0.05), suggesting that these relationships did not depart from linearity. Sensitivity analysis proved our results to be robust.

## 4. Discussion

The purpose of this study was to examine the connection between SII and thyroid function using weighted multivariable linear regression. In this cross-sectional study, we included 6,875 adults (age ≥ 20 years) in which negative correlations of lnSII with FT3 and FT3/FT4 and positive correlations of lnSII with TT4 and TG were found to be statistically significant (*p* < 0.05). We performed subgroup analyses grouped by sex, BMI, and UIC. The results showed that in female participants, FT3 and FT3/FT4 decreased more with increasing lnSII. In the overweight and obese population, the relationship between lnSII and various thyroid functions was more pronounced and statistically significant. The positive correlation between TG and lnSII was only significant in those with a BMI ≥ 30 kg/m^2^. The association between lnSII and thyroid function was only significant in the population with normal iodine nutritional status (UIC ≥ 100 and <300 *µ*g/L). Interaction tests showed that the association between FT3 and lnSII interacted across subgroups. Multiple thyroid functions interacted in the obesity subgroup. Weighted RCS analysis showed a nonlinear relationship between FT3 and lnSII, with no deviation from linearity between TT4, FT3/FT4, TG, and lnSII. Multigroup sensitivity analyses demonstrated the robustness of our results. Our results indicated that SII has an important effect on several thyroid functions.

To our knowledge, this research is the first to investigate the degree of inflammation and thyroid function in vivo. Numerous studies have shown that the inflammatory response has a tremendous impact on the thyroid gland and that inflammation affects the structure of the thyroid gland. Liu et al. [10] found that thyroid nodule formation and progression may be influenced by systemic inflammation, and inflammatory markers are strongly related to the existence and size of thyroid nodules. Inflammation affected thyroid function more. Cross-sectional studies have found that proinflammatory dietary patterns are associated with higher TT4 levels [32]. C-reactive protein (CRP) is a commonly used clinical marker of nonspecific inflammation, and higher CRP levels are independently associated with lower FT3 [29, 33]. Lania et al. [34] reported that the proinflammatory cytokine IL-6 was positively associated with the risk of thyrotoxicosis in a multivariate analysis. These two studies suggest a strong link between systemic inflammation and thyroid hormone levels. Inflammatory responses due to infection are also closely linked to fluctuations in thyroid hormones. A meta-analysis based on 20 studies found that the higher the severity level of COVID-19, the greater the decrease in FT3, FT4, and TSH levels, which could be caused by the “cytokine storm” brought on by the SARS-COV-2 infection [11, 35]. At the same time, infections are environmental triggers for subsequent autoimmune thyroid diseases (AITD) and thyroid dysfunction [36]. IL-21 and IL-21R expression is upregulated in AITD, predominantly Graves' disease and Hashimoto's thyroiditis, and may be involved in the pathogenesis of AITD by enhancing the aberrant immune cascade, which indirectly affects thyroid function [37].

Fluctuations in thyroid hormones also have a significant impact on the inflammatory response in the body. According to Perrotta et al. [38], in vivo experiments demonstrated a significant protective effect of T3 against LPS-induced endotoxemia in mice, and in vitro experiments demonstrated this negative effect on inflammation. Thyroid hormones have a protective effect on the inflammatory process by controlling macrophage maturation and function. Contreras-Jurado et al. [39] found that thyroid hormone receptors act as potent modulators of inflammatory responses and immune homeostasis during sepsis and can directly inhibit cellular responses to inflammatory mediators. Thyroid hormone T3 inhibits IL-6 signaling in macrophages and hepatocellular carcinoma cells, suppresses STAT3 activation, and prevents the deleterious effects of excess hormone signaling during infection [39]. Various pieces of evidence have demonstrated the interaction between inflammation and thyroid function. The SII, as an indicator reflecting systemic inflammatory activity, may be able to predict changes in thyroid hormones to some extent.

The results of subgroup analyses showed that the effect of lnSII on FT3, TT4, and FT3/FT4 was more pronounced in overweight and obese populations with a BMI > 25.0 and <30.0 kg/m^2^ than in the general population. In accordance with our findings, numerous prior studies have demonstrated that obesity and overweight are associated with fluctuations in thyroid function. The values of FT3/FT4 reflect the activity of peripheral T4 to T3 conversion. Zupo et al. [40] found that increased skeletal muscle mass in obesity led to increased T4 to T3 conversion, especially in men. This difference may be mediated by leptin. Obese patients have been found to have high levels of circulating leptin [41, 42]. It has been found that leptin is secreted by adipocytes and promotes the expression of the gene for the thyrotropin-releasing hormone directly in the paraventricular nucleus, eventually stimulating the synthesis and secretion of TSH [43, 44]. Leptin also promotes the conversion of T4 to T3 via deiodinase, which in turn promotes an increase in FT3 [43]. Obesity or overweight affects peripheral T4 to T3 conversion activity and increases the effect of inflammation on conversion activity. Thyroglobulin is the proteogen of thyroid hormones [45], which is strongly linked to diseases such as differentiated thyroid cancer, subacute thyroiditis, and Graves' disease. Our results suggested that the positive correlation between TG and lnSII was significant in those with their BMI ≥30 kg/m^2^ and that obesity may play a role in this positive correlation. Consistent with our results, Guo et al. [46] found that obesity may exacerbate the effects of autoimmunity on TSH and that there is an interaction between obesity and autoimmunity on thyroid function. Fierabracci et al. [47] reported a high prevalence of autoimmune thyroiditis in obese patients. Increased BMI is associated with adipocyte and lymphocyte infiltration of the thyroid gland, which may provide an explanation for the impairment of thyroid function [48]. Obesity is characterized by systemic tissue-specific chronic inflammation and oxidative stress [49, 50], and this environmental alteration may promote the development of AITD and exacerbate the effects of inflammation on the thyroid. The role that obesity plays in the association between inflammation and TG in vivo needs to be demonstrated by more studies.

A close relationship between SII and thyroid function in adults was demonstrated, and SII had a higher sensitivity as a predictor in obese and overweight populations. Interaction tests in the BMI subgroup analyses showed that there was an interaction of FT3, FT3/FT4, and TG with lnSII in the obesity subgroup. This indicated significant differences in the effects of SII on various thyroid functions in obese populations compared to the populations with other BMIs. Several previous studies have proved that obesity and overweight have a significant impact on inflammation and thyroid function. Our study suggested that obesity may play a mediating role in the link between SII and thyroid function, but this hypothesis still needs to be proved by more prospective experiments.

Subgroup analyses based on gender showed more significant associations between FT3, FT3/FT4, and lnSII in women compared with men and the general population. Mendelian randomization studies have found that higher levels of testosterone and estradiol in women are linked to the genetic risk score of FT4, genetically predicting that thyroid function is associated with sex hormones [51]. Estrogen therapy increases the concentration of thyroid hormone-binding globulin. Differentiated thyroid cancer is more prevalent in women than in men, and estrogen may be involved in thyroid changes by regulating enzymatic mechanisms in thyroid cells and inflammatory processes associated with tumor growth [52]. These studies provided a possible explanation for the differences in the effects of SII on thyroid function in different sexes.

There are some strengths and shortcomings in this study. We used a sizable, nationally representative sample for our investigation and considered a variety of potential confounders. The conclusions are highly generalizable. A multigroup sensitivity analysis proved the robustness of the conclusions. However, we recognized some limitations of this study. A variety of evidence has confirmed the interaction between inflammation and thyroid function, but as a cross-sectional study, we cannot draw inferences about causality. We excluded pregnant women and minor participants due to uncertainty about the effects of pregnancy and childhood on thyroid function, and future studies with pregnant women and minors should be conducted. The NHANES database lacks information on medications that could potentially impact thyroid function, and future studies should incorporate this influence. Our model has adjusted for a variety of potential confounders, but it is still unable to completely exclude the effects of other potential confounding variables. More large-scale prospective studies exploring the causal connection between SII and thyroid function are needed in the future.

## 5. Conclusions

In summary, our study identified significant negative correlations between SII and FT3 and FT3/FT4, and a significant positive correlation between SII and TT4. Furthermore, the association between SII and thyroid function was consistent across gender, BMI, and iodine nutritional status, but was more pronounced in women and overweight or obese populations. There were some interactions between this association in obese and overweight populations. Thus, inflammatory activity in the body may have a complex impact on thyroid disease risk, in which obesity may play an important role. Our study suggested that some measures to control the degree of inflammation in the body may be essential to maintaining normal thyroid function in our lives. Besides, to confirm this inference and validate the causal association between SII and thyroid function, additional large-scale prospective investigations are required.

## Figures and Tables

**Table 1:** Baseline characteristics of the NHANES (2007–2012) study population in SII tertiles.

Characteristics	Total	Systemic immune-inflammatory index			*p*-Value
		Tertile 1 (13.750–387.529)	Tertile 2 (387.545–596.077)	Tertile 3 (596.111–4085.000)	
Numbers	6,875	2,292	2,291	2,292	
Age (years)	46.87 ± 0.40	46.04 ± 0.55	47.07 ± 0.47	47.45 ± 0.53	0.064
Sex					**<0.001**
Male	49.81 (48.69, 50.93)	53.86 (51.09, 56.60)	50.57 (47.82, 53.32)	45.22 (42.90, 47.57)	
Female	50.19 (49.07, 51.32)	46.14 (43.40, 48.91)	49.43 (46.68, 52.18)	54.78 (52.44, 57.10)	
Race					**<0.001**
Mexican American	7.75 (5.98, 9.98)	7.77 (6.12, 9.81)	8.02 (6.02, 10.61)	7.45 (5.47, 10.06)	
Non-Hispanic Black	9.87 (7.72, 12.53)	14.50 (11.38, 18.31)	7.95 (6.20, 10.15)	7.42 (5.58, 9.81)	
Non-Hispanic White	71.74 (66.99, 76.05)	67.75 (62.74, 72.39)	72.23 (67.49, 76.53)	75.00 (69.67, 79.66)	
Other Hispanic	4.87 (3.52, 6.70)	4.68 (3.45, 6.33)	5.22 (3.68, 7.34)	4.70 (3.17, 6.90)	
Other race	5.78 (4.75, 7.02)	5.30 (4.00, 6.98)	6.58 (5.11, 8.43)	5.44 (4.15, 7.11)	
Marital status					**0.006**
Nonsingle	64.14 (61.18, 67.00)	66.31 (62.70, 69.74)	65.18 (61.40, 68.78)	61.05 (57.59, 64.39)	
Single	35.86 (33.00, 38.82)	33.69 (30.26, 37.30)	34.82 (31.22, 38.60)	38.96 (35.61, 42.41)	
Education level					0.221
<High school	5.77 (4.94, 6.72)	5.79 (4.83, 6.93)	6.11 (5.02, 7.43)	5.39 (4.38, 6.63)	
Graduated from high school	35.82 (32.65, 39.11)	34.11 (30.50, 37.90)	35.28 (32.30, 38.39)	37.96 (33.04, 43.14)	
College education or above	58.42 (54.84, 61.92)	60.11 (56.24, 63.85)	58.61 (54.94, 62.18)	56.65 (51.40, 61.75)	
Family PIR	3.05 ± 0.06	3.07 ± 0.07	3.14 ± 0.07	2.93 ± 0.08	**0.010**
Family PIR groups					**0.004**
<1	13.85 (12.06, 15.86)	13.90 (11.95, 16.11)	13.28 (11.19, 15.68)	14.39 (12.21, 16.88)	
≥1, <3	35.61 (32.73, 38.60)	35.33 (31.71, 39.13)	32.94 (29.66, 36.40)	38.54 (34.96, 42.26)	
≥3	50.54 (46.80, 54.28)	50.77 (46.60, 54.93)	53.78 (49.45, 58.05)	47.07 (42.36, 51.83)	
Alcohol use					0.406
Never	10.38 (9.10, 11.82)	11.26 (9.32, 13.55)	9.21 (7.91, 10.70)	10.73 (9.00, 12.75)	
Former	16.14 (14.15, 18.35)	14.79 (13.07, 16.70)	16.43 (13.77, 19.48)	17.12 (14.36, 20.29)	
Mild	34.54 (31.96, 37.22)	34.76 (31.39, 38.28)	35.62 (32.53, 38.82)	33.26 (29.23, 37.54)	
Moderate	16.90 (15.33, 18.60)	17.55 (15.21, 20.17)	17.23 (15.33, 19.31)	15.95 (13.29, 19.03)	
Heavy	22.04 (20.47, 23.69)	21.64 (19.21, 24.28)	21.52 (19.35, 23.86)	22.94 (20.70, 25.35)	
Smoking status					**0.002**
Never	53.26 (50.95, 55.57)	56.11 (53.62, 58.57)	53.32 (50.17, 56.44)	50.53 (46.37, 54.68)	
Former	25.19 (23.86, 26.55)	24.46 (22.18, 26.88)	26.84 (24.57, 29.24)	24.21 (22.12, 26.43)	
Now	21.55 (19.58, 23.66)	19.44 (16.76, 22.42)	19.84 (17.41, 22.52)	25.26 (22.13, 28.67)	
BMI (kg/m^2^)	28.67 ± 0.11	27.87 ± 0.19	28.69 ± 0.16	29.40 ± 0.20	**<0.001**
BMI groups					**0.010**
≤18.5	1.54 (1.15, 2.06)	2.06 (1.36, 3.11)	1.22 (0.78, 1.92)	1.37 (0.88, 2.12)	
>18.5, ≤25.0	30.01 (28.35, 31.71)	32.43 (29.64, 35.34)	28.59 (25.92, 31.42)	29.15 (26.33, 32.14)	
>25.0, ≤30.0	34.15 (32.47, 35.87)	35.19 (32.13, 38.38)	35.13 (32.33, 38.03)	32.19 (29.42, 35.09)	
>30	34.31 (32.57, 36.08)	30.33 (27.20, 33.65)	35.06 (32.61, 37.59)	37.29 (34.10, 40.59)	
Diabetes					0.283
No	86.34 (84.95, 87.62)	86.86 (85.15, 88.40)	86.91 (85.02, 88.60)	85.27 (82.85, 87.40)	
Yes	13.66 (12.38, 15.05)	13.14 (11.60, 14.85)	13.09 (11.40, 14.98)	14.73 (12.60, 17.15)	
UIC (*µ*g/L)	382.75 ± 122.44	252.37 ± 35.44	222.54 ± 11.42	666.56 ± 357.35	0.290
UIC groups					0.560
<100	32.60 (30.96, 34.29)	33.47 (30.45, 36.63)	32.35 (29.35, 35.50)	32.04 (29.64, 34.55)	
≥100, <300	48.39 (46.31, 50.48)	49.10 (46.37, 51.84)	47.63 (44.28, 50.99)	48.48 (45.83, 51.14)	
≥300	19.01 (17.23, 20.92)	17.43 (15.44, 19.62)	20.02 (16.61, 23.94)	19.47 (17.70, 21.38)	
HbA1c (%)	5.60 ± 0.02	5.57 ± 0.02	5.60 ± 0.02	5.62 ± 0.03	0.238
ALT (IU/L)	26.19 ± 0.26	26.83 ± 0.48	26.46 ± 0.50	25.32 ± 0.47	0.071
AST (IU/L)	26.21 ± 0.26	27.25 ± 0.50	25.92 ± 0.43	25.51 ± 0.40	**0.022**
Cr (mg/dL)	0.88 ± 0.00	0.88 ± 0.01	0.88 ± 0.01	0.87 ± 0.01	0.383
TC (mmol/L)	5.10 ± 0.02	5.02 ± 0.03	5.16 ± 0.03	5.11 ± 0.03	**0.009**
FT3 (pg/mL)	3.18 ± 0.01	3.21 ± 0.01	3.18 ± 0.01	3.16 ± 0.01	**0.002**
FT4 (ng/dL)	0.80 ± 0.01	0.80 ± 0.01	0.79 ± 0.01	0.81 ± 0.01	**0.044**
TT3 (nmol/L)	1.79 ± 0.01	1.80 ± 0.02	1.80 ± 0.02	1.77 ± 0.02	0.139
TT4 (*µ*g/dL)	7.84 ± 0.04	7.74 ± 0.06	7.79 ± 0.04	7.97 ± 0.05	**<0.001**
TSH (*μ*IU/mL)	2.04 ± 0.06	2.03 ± 0.04	2.03 ± 0.12	2.06 ± 0.09	0.925
FT3/FT4	4.11 ± 0.03	4.14 ± 0.03	4.13 ± 0.03	4.06 ± 0.03	**0.016**
TG (ng/mL)	16.14 ± 0.57	14.83 ± 0.57	15.01 ± 0.81	18.52 ± 1.39	0.062
TGAb (IU/mL)	9.91 ± 1.04	11.19 ± 2.75	11.95 ± 2.58	6.65 ± 1.20	**0.027**
TPOAb (IU/mL)	21.65 ± 1.24	19.47 ± 2.12	20.31 ± 2.27	25.04 ± 2.64	0.236

*Note*: Continuous variables were presented as mean ± SEM, *p*-value was calculated by survey-weighted linear regression analysis. Categorical variables were presented as the percentage (95% confidence interval), *p*-value was calculated by survey-weighted chi-square test. NHANES, National Health and Nutrition Examination Survey; PIR, poverty income ratio; BMI, body mass index; UIC, urine iodine concentration; HbA1c, glycated hemoglobin; ALT, alanine aminotransferase; AST, aspartate aminotransferase; Cr, creatinine; TC, total cholesterol; FT3, free triiodothyronine; FT4, free thyroxine; TT3, total triiodothyronine; TT4, total thyroxine; TSH, thyroid-stimulating hormone; TG, thyroglobulin; TGAb, thyroglobulin antibodies; and TPOAb, thyroid peroxidase antibodies. Bold values signify the statistically significant variables clearly distinguishable from those that are not.

**Table 2:** Weighted univariate linear regression analysis between lnSII and thyroid function.

Thyroid function	lnSII			
	Continuous	Categorical		
		Tertile 1	Tertile 2	Tertile 3
	*β* (95% CI), *p*-value	*β* (95% CI), *p*-value		
FT3	−0.0603 (−0.1078, −0.0128) ^*∗*^	Reference	−0.0242 (−0.0588, 0.0104)	−0.0535 (−0.0851, −0.0218) ^*∗*^ ^*∗*^
FT4	0.0072 (−0.0029, 0.0173)	Reference	−0.0053 (−0.0151, 0.0046)	0.0068 (−0.0039, 0.0175)
TT3	−0.0273 (−0.0559, 0.0013)	Reference	−0.0009 (−0.0349, 0.0332)	−0.0325 (−0.0660, 0.0010)
TT4	0.1837 (0.0974, 0.2700) ^*∗*^ ^*∗*^ ^*∗*^	Reference	0.0526 (−0.0504, 0.1555)	0.2267 (0.1199, 0.3334) ^*∗*^ ^*∗*^ ^*∗*^
TSH	0.0108 (−0.0930, 0.1146)	Reference	0.0012 (−0.2306, 0.2330)	0.0330 (−0.1330, 0.1989)
FT3/FT4	−0.0918 (−0.1658, −0.0178) ^*∗*^	Reference	−0.0078 (−0.0583, 0.0427)	−0.0761 (−0.1349, −0.0172) ^*∗*^
TG	1.8916 (−0.0697, 3.8529)	Reference	0.1784 (−1.6907, 2.0476)	3.6951 (0.6535, 6.7366) ^*∗*^
TPOAb	5.1330 (0.4263, 9.8398) ^*∗*^	Reference	0.8370 (−6.0170, 7.6911)	5.5685 (−1.4221, 12.5590)
TGAb	−3.2196 (−8.6547, 2.2154)	Reference	0.7632 (−8.0861, 9.6124)	−4.5393 (−10.4229, 1.3444)

*Note*: FT3, free triiodothyronine; FT4, free thyroxine; TT3, total triiodothyronine; TT4, total thyroxine; TSH, thyroid-stimulating hormone; TG, thyroglobulin; TGAb, thyroglobulin antibodies; and TPOAb, thyroid peroxidase antibodies.  ^*∗*^*p* < 0.05,  ^*∗*^ ^*∗*^*p* < 0.01,  ^*∗*^ ^*∗*^ ^*∗*^*p* < 0.001.

**Table 3:** Weighted multivariate linear regression analysis between lnSII and thyroid function.

	Model 1^a^*β* (95% CI), *p*-value	Model 2^b^*β* (95% CI), *p*-value	Model 3^c^*β* (95% CI), *p*-value
FT3			
lnSII	−0.0603 (−0.1078, −0.0128) ^*∗*^	−0.0496 (−0.0991, −0.0002)	−0.0559 (−0.1060, −0.0059) ^*∗*^
Tertile 1	Reference	Reference	Reference
Tertile 2	−0.0242 (−0.0588, 0.0104)	−0.0131 (−0.0467, 0.0205)	−0.0180 (−0.0532, 0.0172)
Tertile 3	−0.0535 (−0.0851, −0.0218) ^*∗*^ ^*∗*^	−0.0321 (−0.0626, −0.0015) ^*∗*^	−0.0385 (−0.0689, −0.0082) ^*∗*^
* p* for trend	**0.002**	**0.044**	**0.019**
TT4			
lnSII	0.1837 (0.0974, 0.2700) ^*∗*^ ^*∗*^ ^*∗*^	0.1692 (0.0873, 0.2510) ^*∗*^ ^*∗*^ ^*∗*^	0.1499 (0.0722, 0.2276) ^*∗*^ ^*∗*^
Tertile 1	Reference	Reference	Reference
Tertile 2	0.0526 (−0.0504, 0.1555)	0.0395 (−0.0650, 0.1439)	0.0404 (−0.0656, 0.1464)
Tertile 3	0.2267 (0.1199, 0.3334) ^*∗*^ ^*∗*^ ^*∗*^	0.1885 (0.0850, 0.2921) ^*∗*^ ^*∗*^	0.1637 (0.0638, 0.2636) ^*∗*^ ^*∗*^
* p* for trend	**<0.001**	**<0.001**	**0.004**
FT3/FT4			
lnSII	−0.0918 (−0.1658, −0.0178) ^*∗*^	−0.0724 (−0.1477, 0.0029)	−0.0920 (−0.1667, −0.0173) ^*∗*^
Tertile 1	Reference	Reference	Reference
Tertile 2	−0.0078 (−0.0583, 0.0427)	0.0130 (−0.0376, 0.0635)	−0.0119 (−0.0635, 0.0396)
Tertile 3	−0.0761 (−0.1349, −0.0172) ^*∗*^	−0.0403 (−0.0980, 0.0175)	−0.0635 (−0.1191, −0.0078) ^*∗*^
* p* for trend	**0.014**	0.174	**0.034**
TG			
lnSII	1.8916 (−0.0697, 3.8529)	2.1148 (0.1579, 4.0716)	1.6395 (−0.1964, 3.4754)
Tertile 1	Reference	Reference	Reference
Tertile 2	0.1784 (−1.6907, 2.0476)	0.5005 (−1.3938, 2.3949)	0.1917 (−1.7884, 2.1718)
Tertile 3	3.6951 (0.6535, 6.7366) ^*∗*^	3.7026 (0.6588, 6.7463) ^*∗*^	3.1108 (0.2400, 5.9817) ^*∗*^
* p* for trend	**0.021**	**0.022**	**0.045**
TPOAb			
lnSII	5.1330 (0.4263, 9.8398) ^*∗*^	3.7777 (−1.0059, 8.5613)	4.0172 (−0.5236, 8.5581)
Tertile 1	Reference	Reference	Reference
Tertile 2	0.8370 (−6.0170, 7.6911)	−0.3275 (−7.6876, 7.0326)	−0.7054 (−8.0546, 6.6439)
Tertile 3	5.5685 (−1.4221, 12.5590)	3.7000 (−3.4980, 10.8980)	3.6992 (−3.0884, 10.4868)
* p* for trend	0.123	0.313	0.288

*Note*: FT3, free triiodothyronine; FT4, free thyroxine; TT3, total triiodothyronine; TT4, total thyroxine; TG, thyroglobulin; TGAb, thyroglobulin antibodies; and TPOAb, thyroid peroxidase antibodies. ^a^Model 1: no covariates were adjusted. ^b^Model 2: adjusted for age, sex, race, marital status, family poverty income ratio, and education level. ^c^Model 3: adjusted for age, sex, race, marital status, family poverty income ratio, education level, body mass index, smoking status, alcohol use, diabetes, glycated hemoglobin, alanine aminotransferase, aspartate aminotransferase, creatinine, urine iodine concentration, and total cholesterol.  ^*∗*^*p* < 0.05,  ^*∗*^ ^*∗*^*p* < 0.01,  ^*∗*^ ^*∗*^ ^*∗*^*p* < 0.001. Bold values signify the statistically significant variables clearly distinguishable from those that are not.

**Table 4:** Association between lnSII and thyroid function after subgroup analysis by sex.

Outcomes	Male	Female	*p* for interaction
	*β* (95% CI), *p*-value		
FT3	−0.0367 (−0.0699, −0.0035) ^*∗*^	−0.1142 (−0.1484, −0.0800) ^*∗*^ ^*∗*^ ^*∗*^	**0.001**
TT4	0.1320 (0.0321, 0.2318) ^*∗*^ ^*∗*^	0.1473 (0.0444, 0.2502) ^*∗*^ ^*∗*^	0.832
FT3/FT4	−0.0971 (−0.1698, −0.0243) ^*∗*^ ^*∗*^	−0.1295 (−0.2044, −0.0545) ^*∗*^ ^*∗*^ ^*∗*^	0.539
TG	0.9036 (−1.7166, 3.5238)	1.8839 (−0.8149, 4.5826)	0.605

*Note*: FT3, free triiodothyronine; FT4, free thyroxine; TT3, total triiodothyronine; TT4, total thyroxine; TG, thyroglobulin; TGAb, thyroglobulin antibodies.  ^*∗*^*p* < 0.05,  ^*∗*^ ^*∗*^*p* < 0.01,  ^*∗*^ ^*∗*^ ^*∗*^*p* < 0.001. Bold value signifies the statistically significant variables clearly distinguishable from those that are not.

**Table 5:** Association between lnSII and thyroid function after subgroup analysis by BMI.

Outcomes	≤18.5 kg/m^2^	>18.5, ≤25.0 kg/m^2^	>25.0, ≤30.0 kg/m^2^	>30 kg/m^2^	*p* for interaction
	*β* (95% CI), *p*-value				
FT3	0.0243 (−0.1145, 0.1631)	−0.0277 (−0.0708, 0.0153)	−0.1536 (−0.1948, −0.1125) ^*∗*^ ^*∗*^ ^*∗*^	−0.0417 (−0.0822, −0.0012) ^*∗*^	**<0.001**
TT4	0.1705 (−0.2478, 0.5889)	0.1603 (0.0306, 0.2899) ^*∗*^	0.1939 (0.0699, 0.3179) ^*∗*^ ^*∗*^	0.0857 (−0.0364, 0.2078)	0.659
FT3/FT4	0.0235 (−0.2805, 0.3276)	−0.0554 (−0.1496, 0.0388)	−0.2245 (−0.3146, −0.1344) ^*∗*^ ^*∗*^ ^*∗*^	−0.0588 (−0.1475, 0.0299)	**0.021**
TG	−1.1853 (−12.1406, 9.7699)	0.1532 (−3.2409, 3.5472)	−1.2561 (−4.5032, 1.9909)	5.1916 (1.9946, 8.3887) ^*∗*^ ^*∗*^	**0.031**

*Note*: FT3, free triiodothyronine; FT4, free thyroxine; TT3, total triiodothyronine; TT4, total thyroxine; TG, thyroglobulin; and TGAb, thyroglobulin antibodies.  ^*∗*^*p* < 0.05,  ^*∗*^ ^*∗*^*p* < 0.01,  ^*∗*^ ^*∗*^ ^*∗*^*p* < 0.001. Bold values signify the statistically significant variables clearly distinguishable from those that are not.

**Table 6:** Association between lnSII and thyroid function after subgroup analysis by UIC.

Outcomes	<100 *µ*g/L	≥100, <300 *µ*g/L	≥300 *µ*g/L	*p* for interaction
	*β* (95% CI), *p*-value			
FT3	−0.0343 (−0.0771, 0.0084)	−0.1125 (−0.1463, −0.0788) ^*∗*^ ^*∗*^ ^*∗*^	−0.0412 (−0.0940, 0.0115)	**0.007**
TT4	0.1087 (−0.0200, 0.2373)	0.1737 (0.0721, 0.2753) ^*∗*^ ^*∗*^ ^*∗*^	0.1016 (−0.0571, 0.2603)	0.639
FT3/FT4	−0.0801 (−0.1738, 0.0136)	−0.1481 (−0.2221, −0.0741) ^*∗*^ ^*∗*^ ^*∗*^	−0.0796 (−0.1952, 0.0360)	0.428
TG	−0.6181 (−3.9921, 2.7558)	2.9681 (0.3038, 5.6324) ^*∗*^ ^*∗*^	0.5213 (−3.6415, 4.6840)	0.229

*Note*: FT3, free triiodothyronine; FT4, free thyroxine; TT3, total triiodothyronine; TT4, total thyroxine; TG, thyroglobulin; and TGAb, thyroglobulin antibodies.  ^*∗*^*p* < 0.05,  ^*∗*^ ^*∗*^*p* < 0.01,  ^*∗*^ ^*∗*^ ^*∗*^*p* < 0.001. Bold value signifies the statistically significant variables clearly distinguishable from those that are not.

## Data Availability

This study used data from a publicly available datasets. These data can be found at https://www.cdc.gov/nchs/nhanes.
